# Evaluation of the Antibacterial and Anti-Inflammatory Effects of a Natural Products-Containing Toothpaste

**DOI:** 10.3389/fcimb.2022.827643

**Published:** 2022-02-10

**Authors:** Cai Qi, Xian Peng, Shaotang Yuan, Miaomiao Zhang, Xin Xu, Xingqun Cheng

**Affiliations:** ^1^The State Key Laboratory of Oral Diseases and National Clinical Research Center for Oral Diseases, West China Hospital of Stomatology, Sichuan University, Chengdu, China; ^2^Department of Cariology and Endodontics, West China Hospital of Stomatology, Sichuan University, Chengdu, China; ^3^MHOME (Guangzhou) Industrial Co., Ltd, Guangzhou, China; ^4^Department of Geriatric Dentistry, West China Hospital of Stomatology, Sichuan University, Chengdu, China

**Keywords:** natural products, antibacterial activity, anti-inflammatory effect, oral diseases, fluoride, toothpaste

## Abstract

Fluoride-containing toothpaste is daily used in toothbrush. Some compounds derived from natural herbs that have antibacterial and anti-inflammatory activities has attracted increasing attention as potential supplements for the control of oral diseases. In this paper, a natural product mixture (NPM-8) containing eight herbs extracts was added to toothpaste, and its antibacterial and anti-inflammatory effects were investigated. The results showed that NPM-8-containing toothpaste exhibited superior and faster inhibitory and bactericidal effects against *S. mutans*, *S. sanguinis* and *P. gingivalis* than that of the NPM-8-free toothpaste. NPM-8-containing toothpaste significantly reduced the biomass of single-species or three-species biofilms. The cytotoxicity of the NPM-8-containing toothpaste was similar to that of the conventional fluoride toothpaste and CHX. The NPM-8-containing toothpaste could significantly inhibit IL-1β and IL-6 production in HGE cells and exhibited a better anti-inflammatory effect than that of the NPM-8-free toothpaste. In conclusion, NPM-8-containing fluoride toothpaste is superior to conventional fluoride toothpaste in regard to their antibacterial, antibiofilm, and anti-inflammatory properties. NPM-8-containing toothpaste also has good biocompatibility and is safe for daily use. It indicates that NPM-8 is a promising natural product mixture in oral health.

## Introduction

Oral diseases are common diseases that occur frequently and are estimated to affect half of the global population (James et al., 2018; Zhou et al., 2018). The occurrence and development of most oral diseases are closely associated with oral microbiome (Marsh, 2005). Approximately 700 species of oral bacteria, fungi, and other oral microorganisms are embedded in extracellular matrix, functionally and structurally adhering to the tooth surface or the oral mucosa in the form of biofilms (Marsh and Zaura, 2017). Commensal *Streptococcus sanguinis*, as one of the pioneer colonizers, can produce hydrogen peroxide, mediating microbial interactions and modifying the spatial-temporal arrangement of the species in dental plaque (Cheng et al., 2020). *Streptococcus mutans*, which has acidogenic/aciduric characteristics and the ability to produce extracellular matrix, is considered the primary etiological agent of dental caries (Klein et al., 2015). *Porphyromonas gingivalis* can produce various virulence factors, such as lipopolysaccharide (LPS), playing important roles in the pathogenesis of gingivitis and periodontitis (Allaker and Douglas, 2009; Xu et al., 2020). Within the oral biofilm, these bacteria can also trigger the host immune response and induce the host to secrete cytokines such as interleukin-1β (IL-1β) and interleukin-6 (IL-6), disrupting host immune homeostasis and causing oral dysbiosis (Hajishengallis and Lamont, 2021).

Therefore, it is very important to control dental plaque formation to maintain oral health. Various mechanical and chemical measures have been developed to control dental plaque, including toothbrush with fluoride-containing toothpaste, floss and mouthwash (van der Weijden and Slot, 2011; Almas and Almas, 2014; Chapple et al., 2015; Figuero et al., 2017). Studies have demonstrated that these daily oral hygiene practices are effective in reducing dental plaque and controlling dental caries and periodontal diseases (Rodrigues et al., 2011). However, in the high-risk group, combinatory application of antimicrobial/anti-inflammatory agents is necessary (Gluzman et al., 2013; Chapple et al., 2015; Figuero et al., 2017; Featherstone and Chaffee, 2018).

Nowadays, compounds derived from natural herbs has attracted increasing attention as potential supplements for the control of oral diseases (Van Leeuwen et al., 2011; Chinsembu, 2016). Natural products with antibacterial and anti-inflammatory effects have been wildly used to control oral infections and promote oral health (Chinsembu, 2016). *Centella asiatica* can inhibit LPS-induced inflammatory response in gingival tissues, and promote type I collagen synthesis and osteogenic differentiation in human periodontal ligament cells (Nowwarote et al., 2013; Hao et al., 2017; Fitri et al., 2018; Soe et al., 2020). Extracts of *Polygonum cuspidatum* root and *Camellia sinensis* leaf inhibit the bacterial adherence and acid and exopolysaccharide (EPS) production of *S. mutans* (Allaker and Douglas, 2009). *Rosmarinus officinalis* and *Salvia officinalis* can inhibit the growth and biofilm formation of oral microorganisms, particularly oral streptococci (Allaker and Douglas, 2009; de Oliveira et al., 2017). The extract of *Glycyrrhiza glabra* has an inhibitory effect on the growth, volatile sulfur compound (VSC) production and protease activity of *P. gingivalis* (Sidhu et al., 2020). Extract of the *Chamomilla recutita* plant was revealed to effectively alleviate gingival bleeding, as the extract has antimicrobial and anti-inflammatory properties comparable to those of chlorhexidine (CHX) (Batista et al., 2014). The extract of *Scutellaria baicalensis* ameliorates the destruction of periodontal ligament *via* inhibition of inflammatory cytokine expression in animal study (Kim et al., 2018).

Studies have found that natural product mixtures are more effective than purified compounds due to beneficial “synergistic” interactions (Stermitz et al., 2000; Junio et al., 2011). Therefore, a natural product mixture of eight ingredients (NPM-8), including *Centella asiatica*, *Polygonum cuspidatum* root, *Scutellaria baicalensis* root, *Camellia sinensis* leaf, *Glycyrrhiza glabra* (*licorice*) root, *Chamomilla recutita* (*Matricaria*) flower, *Rosemary rosmarinus officinalis* leaf and *Salvia officinalis* (*Sage*), was prepared and speculated to inhibit dental plaque formation and alleviate inflammation such as gingival swelling and redness, thereby facilitating the management of dental caries and gingivitis. The current study evaluated the antimicrobial and anti-inflammatory effects of a NPM-8 containing fluoride toothpaste, aiming to provide a promising measure to promote oral health.

## Materials and Methods

### Bacterial Strains and Growth Conditions

*S. mutans* UA159, *S. sanguinis* ATCC10556, and *P. gingivalis* ATCC33277 were obtained from the Stare Key Laboratory of Oral Diseases (Sichuan University, Chengdu, China). *S. mutans*, *S. sanguinis* and *P. gingivalis* were routinely cultured in brain heart infusion (BHI) broth (Difco, Sparks, MD) or on BHI agar plates under anaerobic conditions (80% N_2_, 10% CO_2_, 10% H_2_) at 37°C. A total of 1 μg/mL hemin (Sigma, St. Louis, MO, USA) and 1 μg/mL menadione (Sigma, USA) were added to BHI (designated BHIHM), or 5% demineralized sheep blood was added to BHI agar plates, particularly for *P. gingivalis*. When needed, the medium was supplemented with 1% sucrose (designated BHIHMS).

### Treatment With Test Agents

Test agents included 1× phosphate-buffered saline (PBS) (100 mL of 10× PBS: in distilled water, 1.236% Na_2_HPO_4_, 0.180% NaH_2_PO_4_, 8.5% NaCl, pH=7.4), NPM-8-free toothpaste slurry extract, NPM-8-containing toothpaste slurry extract, and 0.04% CHX. The test toothpastes were provided by MHOME (Guangzhou) Industrial Co., Ltd, China, see **Supplementary Tables 1** and **2** for detailed compositions of the toothpastes used in this study. PBS was the negative control, the two toothpastes were experimental groups, and CHX was the positive control. The test agents were prepared as described in a previous study with some modifications (ISO, 2009; Birant et al., 2021). Briefly, the toothpaste slurry was prepared by mixing 10 g toothpaste into 50 mL extraction solvents (weight:volume = 1:5) under constant stirring. BHI culture medium or Dulbecco’s modified Eagle’s medium (DMEM; GibcoTM, Invitrogen, Carlsbad, CA, USA) served as the extraction solvents. Subsequently, the toothpaste slurry was centrifuged at 4200 rpm for 10 min, and the supernatant of the extracts was obtained to treat the bacteria or cells (ISO 10993‐5%J IOS, 2009). The mucoadhesive polymer of NPM-8-containing toothpaste is xanthan gum (high permeability), which helps control the release of active ingredients during the 120 s (Aspinall et al., 2021).

### Growth Curves Analysis

Overnight (16 h) culture of *S. mutans* and *S. sanguinis* and 48 h culture of *P. gingivalis* were adjusted to 1*10^7^ CFU/mL based on the standard curve (OD_600nm_ versus CFU/mL) of each bacterial species. Bacteria were harvested by centrifugation at 4000 g at 4°C for 10 min and anaerobically incubated in a fresh BHI culture with different test agents at 37°C for 10 h. The OD_600nm_ value was measured by using a spectrophotometer (UV1601, Shimadzu, Japan) every 1 h for 10 h. This experiment was conducted with triplicate samples at each time point and reproduced at least three separate times.

### Time-Dependent Killing Assay

The time-dependent killing assay was carried out as described previously with some modifications (Koo et al., 2002)*. S. mutans*, *S. sanguinis* and *P. gingivalis* were diluted to 1 × 10^7^ CFU/ml in BHI broth containing test agents. At 0, 5, 10, 20, and 30 min, samples were serially diluted in PBS, and 50 μl aliquots were spread onto BHI agar plates (*P. gingivalis* was spread onto BHI blood agar plates). All plates were incubated anaerobically for 48 h followed by enumeration of CFU. Time-kill or dose-dependent killing curves were constructed by plotting lg^CFU/ml^ versus the incubation time or concentration. The experiment was performed in triplicate and repeated at least three times.

### Oxford Cup Bacteriostatic Test

The Oxford cup method was used to determine the diameter of the inhibition zone (Liang et al., 2021). Overnight (16 h) culture of *S. mutans* and *S. sanguinis* and 48 h culture of *P. gingivalis* were adjusted to 1*10^7^ CFU/mL. One milliliter of the three bacteria was added to 45 ml of 55°C BHI solid medium. When it was solidified, sterile Oxford cups (φ=6 mm) were placed on the surface of the plate at a distance of 2 cm, and then 0.2 ml of test agents was added to the Oxford cup. After 48 h of anaerobic incubation, the diameter of bacteriostatic circles was measured with a digital caliper (Digimatic Caliper, Mitutoyo, Japan).

### Biofilm Analysis

For the biofilm formation experiment, the overnight (16 h) culture of *S. mutans* and *S. sanguinis* and 48 h culture of *P. gingivalis* were adjusted to 1*10^7^ CFU/mL and 1:10 diluted in BHIHMS media with test agents. Bacteria were inoculated in a 96-well plate either individually or mixed together (the inoculum ratio of *S. mutans*:*S. sanguinis*:*P. gingivalis* was 1:1:2) to form single-species or three-species biofilms (Zheng et al., 2015). After 24 h of treatment, crystal violet (CV) staining was used to quantify the biomass of the biofilms (Bedran et al., 2014; Stiefel et al., 2016). Briefly, the biofilms were washed three times with sterile PBS, fixed with methanol for 15 min, and then stained with 100 μL 0.1% CV for 10 min at room temperature. The supernatants were gently removed, the wells were rinsed three times with PBS, and the bound CV was dissolved in 100 μL 95% ethanol. The absorbance of the solution was measured at a wavelength of 595 nm by using a spectrophotometer. The experiment was performed in triplicate and repeated at least three times.

For the biofilm removal experiment, bacteria were inoculated in a 96-well plate either individually or mixed together to form single-species or three-species biofilms. After 24 h of incubation, half of the samples were used to crystal violet staining and got the biomass #1. The other half of the pre-formed biofilms were washed twice with PBS, and challenged with the four test agents for additional 24 h (Palmeira-de-Oliveira et al., 2012). Crystal violet staining was used to quantify the biomass of the biofilms and got the biomass #2. The biomass reduction (%) was determined as follows: Biomass reduction (%) = (biomass #1−biomass #2)/biomass #1*100. The experiment was performed in triplicate and repeated at least three times.

### Cell Culture and Treatment

Human gingival epithelial (HGE) cells (Single donor, CELLnTEC, Bern, Switzerland) were identified by morphology and immunohistochemistry. HGE cells are typical keratinocytes with uniform size, compact arrangement. Pan cytokeratin staining of the cells showed positive. HEG cells were grown in DMEM supplemented with 10% fetal bovine serum (FBS; Invitrogen) and 1% penicillin–streptomycin solution (Invitrogen) and cultured at 37°C in a humidified atmosphere containing 5% CO_2_. HGE cells were treated with test agents for 3 min, 5 min, 1 h, and 24 h, and the morphological changes in the cells were observed and recorded by binocular stereoscopic microscopy (Olympus, BH2-RFCA).

### *In Vitro* Cytotoxicity Assay

The cytotoxicity of the toothpaste was evaluated by the Cell Counting Kit-8 (CCK-8; Dojindo, Kumamoto, Japan) assay as described by Park et al. (Park et al., 2014). All cells were inoculated into 96-well plates at a density of 1 × 10^4^ cells per well for 24 h and then treated with test agents for 3 min, 5 min, 1 h, and 24 h. After treatment, the wells were washed with sterile PBS, and fresh media were added. Ten microliters of a CCK-8 solution was added to each well and incubated for 1.5 h. The absorbance of each well was measured at a wavelength of 450 nm against a blank that only contained medium. Cell viability was calculated according to the following formula: % viability = (OD_450nm_ of treated group − OD_450nm_ of blank control)/(OD_450nm_ of negative control − OD_450nm_ of blank control) × 100%. The experiment was conducted in triplicate and repeated three times.

### ELISA

Enzyme-linked immunosorbent assay (ELISA) was performed to quantify LPS production by *P. gingivalis* after the test agent treatment. ELISA was also used to determine the cytokine concentrations of HGE cells subjected to *P. gingivalis* stimulation by using IL-1β and IL-6 ELISA kits (Cloud-clone corp, Wuhan, China). Forty-eight-hour cultures of *P. gingivalis* were adjusted to 1*10^7^ CFU/mL. The tube was centrifuged at 4000 rpm for 10 min, and the supernatant was collected for stimulation. HGE cells were incubated in 6-well plates (Corning, Corning, USA) for 24 h, stimulated with the *P. gingivalis* supernatant for 5 min, and subsequently treated with test agents for 3 or 5 min. After treatment, the cells were cultured in fresh DMEM without test agents for 2 h, and then the cell media were collected for ELISA. Briefly, 100 µL of each sample or standard was added to the appropriate well. Then, 100 µL of antibody cocktail was added to each well. The plate was covered and incubated for 1 h at 37°C. Each well was washed with wash buffer 5 times, 200 µL of tetramethylbenzidine substrate was added to each well, and the plate was incubated for 10 min in the dark. The reaction was terminated by adding Stop Solution to each well. The OD_450nm_ of each well was measured with a spectrophotometer. The experiment was conducted in triplicate and repeated three times.

### Statistical Analysis

All experiments were performed in triplicate and repeated at least three times independently. Statistical analysis of the data was performed with SPSS (version 18.0 for Windows; SPSS Inc., Chicago, IL, USA). One-way analysis of variance and *post hoc* Student-Newman-Keuls test were used to compare the data of two or more groups. Data are presented as the mean ± standard deviation (SD). Data were considered significantly different if the two-tailed *p* value was <0.05.

## Results

### NPM-8-Containing Toothpaste Exhibits a Better Antibacterial Effect Than That of NPM-8-Free Toothpaste

**Figure 1:**
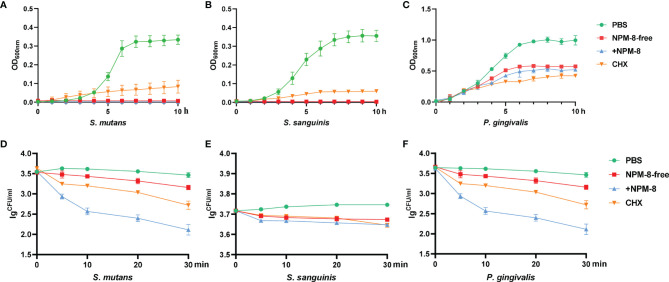
Antibacterial activity of the four test agents on *S. mutans*, *S. sanguinis* and *P. gingivalis*. **(A)** Growth curves of *S. mutans* in PBS, NPM-8-free toothpaste, NPM-8-containing toothpaste and CHX treatment. **(B)** Growth curves of *S. sanguinis* under the four test agent treatments. **(C)** *P. gingivalis* growth curves. **(D)** Time-kill curves for *S. mutans* under the four test agent treatments. **(E)** *S. sanguinis* time-kill curves. **(F)** *P. gingivalis* time-kill curves. Data are represented as means ± SD.

Growth curves were first measured to explore the antibacterial activity of the two toothpastes against oral commensal bacteria, with PBS treatment as a negative control and CHX treatment as a positive control. NPM-8-free toothpaste, NPM-8-containing toothpaste and CHX almost completely inhibited the growth of *S. mutans* and *S. sanguinis* (**Figures 1A, B**). For the above three groups, *P. gingivalis* exhibited a slower growth rate and an extended lag phase and failed to reach the same OD_600nm_ values as that of the PBS treatment group after a 10 h incubation (**Figure 1C**).

A time-dependent killing assay was then performed to evaluate the kinetic-killing effect of the four treatments against *S. mutans*, *S. sanguinis* and *P. gingivalis*. The two toothpastes and CHX displayed significant time-dependent bactericidal effects compared with that of PBS (**Figures 1D–F**). However, the NPM-8-containing toothpaste exhibited the greatest and fastest bactericidal activity, especially against *S. mutans* and *P. gingivalis* (**Figures 1D–F**).

**Figure 2:**
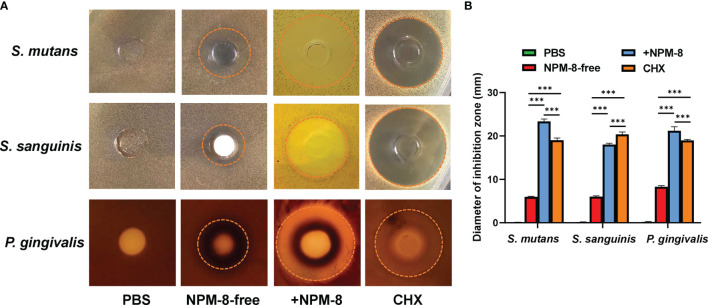
Results of the Oxford cup bacteriostatic test. **(A)** Representative images of the inhibition zone of *S. mutans, S. sanguinis* and *P. gingivalis* treated with PBS, NPM-8-free toothpaste, NPM-8-containing toothpaste and CHX. **(B)** The diameters of the inhibition zones with different test agents against *S. mutans*, *S. sanguinis* and *P. gingivalis*. Data are presented as means ± SD. ***P < 0.001.

The Oxford cup method was further conducted to determine the antibacterial effect of the four test agents. As shown in **Figure 2**, the diameters of the inhibition zones against *S. mutans*, *S. sanguinis* and *P. gingivalis* treated with NPM-8-containing toothpaste were significantly larger than those of the NPM-8-free toothpaste group. Moreover, the NPM-8-containing toothpaste treatment group exhibited significantly larger inhibition zones against *S. mutans* and *P. gingivalis* than those of the CHX group (**Figure 2**). All the results suggest that NPM-8-containing toothpaste has a better antibacterial effect than that of conventional fluoride toothpaste and has comparable or even better antibacterial activity than that of CHX.

### NPM-8-Containing Toothpaste Effectively Inhibits Oral Biofilms

**Figure 3:**
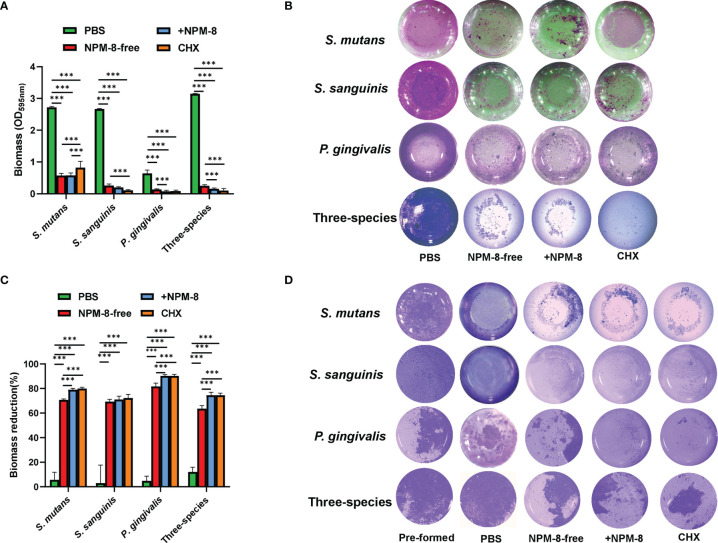
Antibiofilm activity of the four test agents on *S. mutans*, *S. sanguinis* and *P. gingivalis* single-species and three-species biofilms. **(A)** Quantitative analysis of the effect of test agents on the biofilm formation. **(B)** Representative crystal violet staining images of the biofilms. **(C)** Quantitative analysis of biofilm biomass reduction. **(D)** Representative crystal violet staining images of the pre-formed biofilms and the biofilms after treatment. Data are presented as means ± SD. ***P < 0.001.

Crystal violet staining was used to determine the biomass of *S. mutans, S. sanguinis* and *P. gingivalis* single-species and three-species biofilms. For the biofilm formation experiment, the biofilm biomass was significantly decreased after treatment by NPM-8-free toothpaste, NPM-8-containing toothpaste and CHX, as compared to that of the PBS-treated group. More importantly, the NPM-8-containing toothpaste exerted further inhibition on the biofilm formation of *P. gingivalis* and the three-species biofilm compared to NPM-8-free toothpaste (**Figures 3A, B**).

For the biofilm removal test, the biofilm biomass significantly reduced after NPM-8-free toothpaste, NPM-8-containing toothpaste and CHX treatment compared with that of the PBS treatment group. NPM-8-containing toothpaste exhibited significantly higher reduction of *S. mutans*, *P. gingivalis* and three-species biofilm biomass compared with that of the NPM-8-free toothpaste group (**Figures 3C, D**). These results indicate that NPM-8-containing toothpaste can effectively inhibit the biofilm formation and disrupt the established biofilm.

### NPM-8-Containing Toothpaste Shows Similar Cytotoxicity on HGE Cells as Compared to Conventional Fluoride Toothpaste

**Figure 4:**
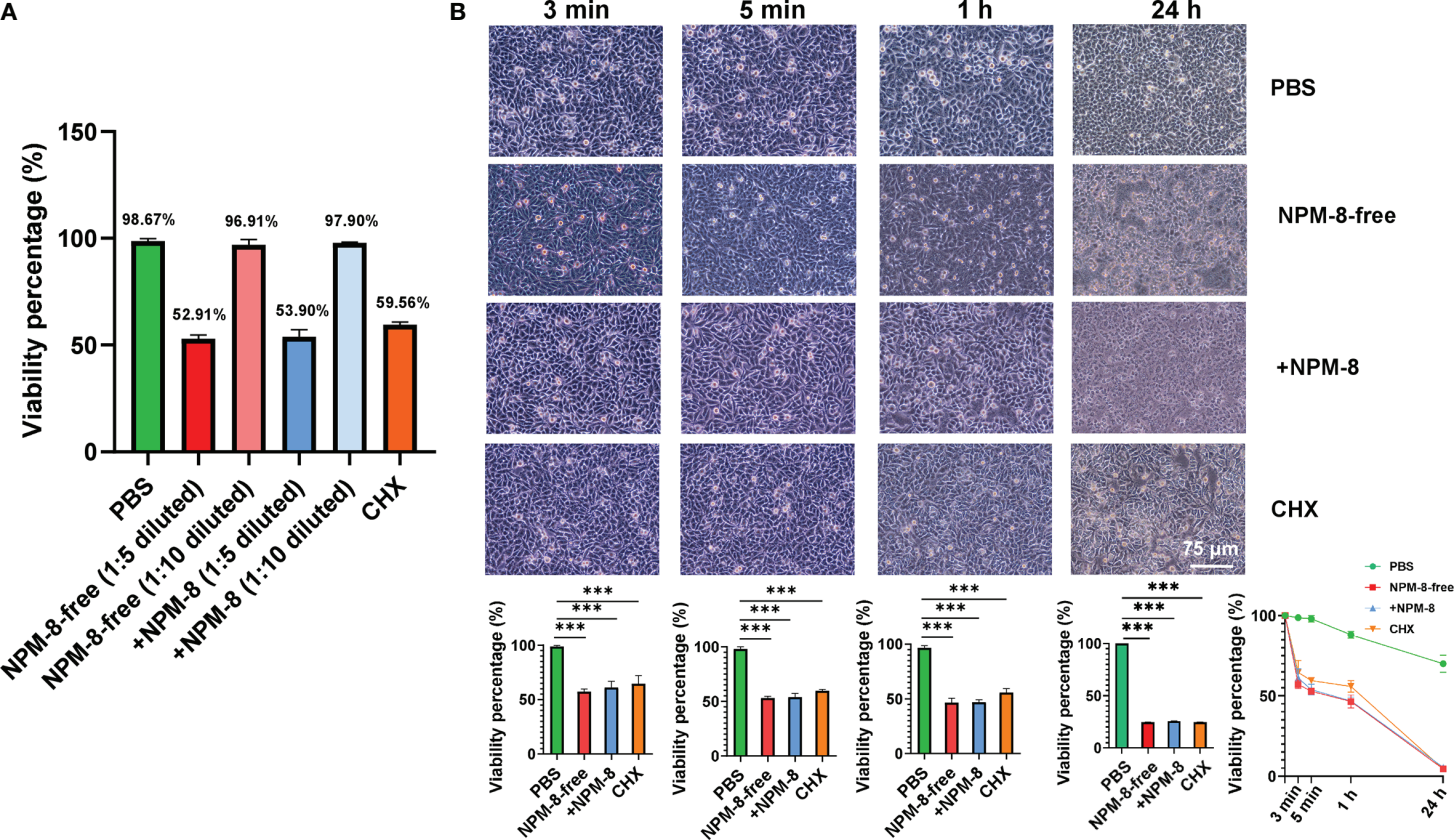
Cytotoxicity of the four test agents on HGE cells. **(A)** Viability of the HGE cells exposed to PBS, 1:5- or 1:10-diluted toothpastes, and CHX treatment. **(B)** Representative images of HGE cells after stimulation with the four test agents (PBS, 1:5 diluted toothpastes, and CHX) for 3 min, 5 min, 1 h and 24 h. The HGE cells viability was quantified. Data are presented as means ± SD. ***P < 0.001.

The two toothpastes were diluted 1:5 or 1:10 with PBS and were stimulated with HGE cells for 5 min. Compared to PBS, the two 1:10 diluted toothpastes exhibited similar cytotoxicity on HGE cells (**Figure 4A**). No significant difference was observed between the two 1:5 diluted toothpastes and CHX groups (**Figure 4A**). Then, the HGE cells were exposed to 1:5 diluted toothpaste stimulation for 3 min, 5 min, 1 h and 24 h, with PBS as the negative control and CHX as the positive control. PBS treatment had no effect on the morphology of HGE cells. HGE cells were lysed after toothpaste slurry and CHX stimulation (**Figure 4B**). The two toothpaste and CHX treatments exhibited time-dependent killing effects on HGE cells (**Figure 4B**). However, for both the short-term (3 min and 5 min) and long-term (1 h and 24 h) stimulations, no significant difference was observed among the three groups (**Figure 4B**). These results suggest that addition of NPM-8 in the toothpaste does not affect the biocompatibility of toothpaste *in vitro*.

### NPM-8-Containing Toothpaste Exhibits Better Anti-Inflammatory Activity Than Conventional Fluoride Toothpaste

**Figure 5:**
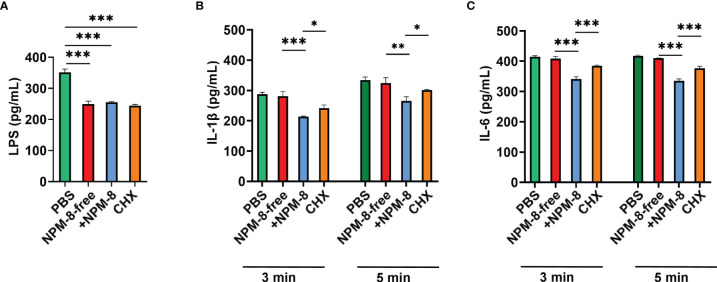
Anti-inflammatory activity of the four test agents. **(A)** Quantification analysis of the LPS produced by *P. gingivalis* after the test agent treatment. **(B)** Quantification analysis of the proinflammatory cytokines IL-1β in HGE cells stimulated with *P. gingivalis* supernatant and subsequently treated with test agents for 3 or 5 min. **(C)** Quantification analysis of the proinflammatory cytokine IL-6 in HGE cells stimulated with *P. gingivalis* supernatant and subsequently treated with test agents for 3 or 5 min. Data are presented as the means ± SD. *P < 0.05, **P < 0.01, ***P < 0.001.

LPS is a critical virulence factor of *P. gingivalis*. The effects of the two toothpastes and CHX on LPS release of *P. gingivalis* were not significantly different (**Figure 5A**). ELISA was used to determine cytokine production by HGE stimulated with *P. gingivalis* supernatant followed by 3 min or 5 min of treatment with the test agents. The IL-1β and IL-6 levels showed no significant change after NPM-8-free toothpaste treatment compared to those of the PBS group (**Figures 5B, C**). NPM-8-containing toothpaste treatment significantly attenuated IL-1β and IL-6 production compared with that of the NPM-8-free toothpaste and CHX treatment groups (**Figures 5B, C**). These results suggest that NPM-8-containing toothpaste can inhibit cytokine production, exhibiting better anti-inflammatory activity than that of conventional fluoride toothpaste.

## Discussion

Most oral diseases, such as dental caries and periodontal diseases, are prevalent chronic noncommunicable diseases associated with dental plaque biofilms. Similar to most chronic diseases, if steps are taken to control the causative factors, the initiation and advancement of dental caries and periodontal diseases can be better controlled. Therefore, daily effective plaque control is indispensable for the management of dental caries and periodontal diseases. Disruption of dental plaque by means of mechanical and chemical toothbrushes is crucial for the maintenance of dental and periodontal health (van der Weijden and Slot, 2011). However, for high-risk population, additional antimicrobial and anti-inflammatory measures are necessary (Chapple et al., 2015; Serrano et al., 2015; Featherstone and Chaffee, 2018). Compounds derived from natural herbs have attracted increasing attentions due to their good antibacterial and anti-inflammatory activities (Van Leeuwen et al., 2011; Chinsembu, 2016). In this paper, NPM-8, a compound extracted from eight herbs, was added to the fluoride toothpaste, and we demonstrated that the NPM-8-containing toothpaste had superior antibacterial and anti-inflammatory activity as compared to the conventional fluoride toothpaste, representing a promising oral hygiene product for the promotion of oral health.

Three representative common bacteria of dental plaque, *S. mutans*, the widely recognized cariogenic bacteria; *S. sanguinis*, the early colonizing bacteria of dental plaque; and *P. gingivalis*, the important periodontal pathogen, were selected for the antibacterial studies. The results derived from growth curves, time-dependent killing assays, and the Oxford cup method indicated that NPM-8-containing toothpaste could effectively inhibit the growth of *S. mutans*, *S. sanguinis*, and *P. gingivalis*, exhibited better and faster antibacterial effects than that of NPM-8-free toothpaste and had comparable or even better antibacterial activity than that of CHX. These experiments verified the antibacterial effect of NPM-8. As expected, many studies have demonstrated the antibacterial activities of the natural compounds contained in NPM-8 (Allaker and Douglas, 2009; de Oliveira et al., 2017; Sidhu et al., 2020).

Oral microorganisms mainly exert their pathogenic effect in the form of dental plaque biofilms in the oral cavity (Bowen et al., 2018). The current study also employed single-species and three-species biofilms consisting of *S. mutans*, *S. sanguinis* and *P. gingivalis* to determine the antibiofilm activity of NPM-8. Of note, both NPM-8 containing and NPM-8-free toothpaste significantly reduced oral biofilms as compared to the PBS-treated control. We speculate that the surfactant contained in the toothpaste may contribute most to the suppression of oral biofilms in this scenario. However, further additive effects on the suppression of oral biofilms by the NPM-8-containing toothpaste was observed as compared to the NPM-8-free toothpaste, particularly for the removal of pre-established biofilms. Considering the antimicrobial effects of NPM-8 on the growth of oral bacteria, we believe that addition of NPM-8 in the toothpaste may further promote oral health, although more data obtained from sophisticated *in vitro* and *in vivo* models are still needed to validate its clinical benefit in the future.

The biocompatibility of a toothpaste is critical for its application. The gingival epithelium is a natural barrier that protects periodontal tissue against latent invasion (Groeger and Meyle, 2015). The present study evaluated the cytotoxicity of NPM-8-containing toothpaste against HGE cells. The NPM-8-containing toothpaste exhibited similar cytotoxicity against HGE cells to that of conventional fluoride toothpaste and CHX, regardless if the toothpaste was diluted to different concentrations and if the toothpaste stimulated the cells for the same time or for different times. This suggests that NPM-8-containing toothpaste is safe for daily use.

The anti-inflammatory activity of the NPM-8-containing toothpaste was further assessed. Bacterial infections and virulence factors produced by bacteria can induce cytokine secretion and trigger the host immune response (Hajishengallis and Lamont, 2021). The oral cavity maintains an ecological equilibrium and is important for overall oral health. The condition of oral equilibrium is reliant upon not only homeostasis within the biofilm but also the balance between the polymicrobial community and the host immune state (Hajishengallis and Lamont, 2021). Therefore, HGE cells were stimulated with the *P. gingivalis* supernatant, followed by treatment with the test agents, and the levels of the inflammatory mediators IL-1β and IL-6 in the cells were determined. NPM-8-containing toothpaste treatment significantly attenuated IL-1β and IL-6 production compared with that of the NPM-8-free toothpaste and CHX treatment groups, suggesting that NPM-8-containing toothpaste has superior anti-inflammatory effects compared with that of conventional fluoride toothpaste. The LPS production of *P. gingivalis* after the test agent treatment was also measured. LPS is the main virulence factor of *P. gingivalis* and could effectively induce the host immune response. The results showed that the effects of the test agents on LPS released by *P. gingivalis* were not significantly different. This further confirmed the anti-inflammatory property of the NPM-8-containing toothpaste. More studies are needed to further explore the anti-inflammatory activity and oral ecological modification effect of NPM-8.

In conclusion, NPM-8-containing fluoride toothpaste is superior to conventional fluoride toothpaste in regard to their antibacterial, antibiofilm, and anti-inflammatory properties. NPM-8-containing toothpaste also has good biocompatibility and is safe for daily use. It indicates that NPM-8 is a promising natural product mixture in oral health.

## Data Availability Statement

The raw data supporting the conclusions of this article will be made available by the authors, without undue reservation.

## Author Contributions

CQ and XC contributed to the conception, design, data acquisition and analysis, and drafted and critically revised the manuscript. XP contributed to the conception, design, and data interpretation and critically revised the manuscript. SY and MZ contributed to the conception, design, and critically revised the manuscript. XX contributed to the conception, design, and data interpretation and critically revised the manuscript. All authors approved the final manuscript and agreed to be accountable for all aspects of the work.

## Funding

This study was supported by the National Natural Science Foundation (82101002 to XC), Technology Innovation Research and Develop Project of Chengdu (2021-YF05-01866-SN to XC) Postdoctoral Science Foundation of Sichuan University (2021SCU12113 to XC), and a Joint Research Program between Sichuan University and MHOME (Guangzhou) Industrial Co., Ltd (19H1091).

## Conflict of Interest

Author SY and MZ is employed by MHOME (Guangzhou) Industrial Co., Ltd.

The remaining authors declare that the research was conducted in the absence of any commercial or financial relationships that could be construed as a potential conflict of interest.

This study received funding from MHOME (Guangzhou) Industrial Co., Ltd. The funder had the following involvement with the study: conception, design, and critically revised the manuscript.

## Publisher’s Note

All claims expressed in this article are solely those of the authors and do not necessarily represent those of their affiliated organizations, or those of the publisher, the editors and the reviewers. Any product that may be evaluated in this article, or claim that may be made by its manufacturer, is not guaranteed or endorsed by the publisher.

## Abbreviations

LPS, lipopolysaccharide; IL-1β, interleukin-1β; IL-6, interleukin-6; NaF, sodium fluoride; NF-κB, nuclear factor kappa-B; EPS, exopolysaccharides; VSCs, volatile sulfur compounds; CHX, chlorhexidine; BHI, brain heart infusion; PBS, phosphate buffered saline; DMEM, Dulbecco’s modified Eagle’s medium; CV, crystal violet; HGE, human gingival epithelial cell; FBS, fetal bovine serum; CCK-8, Cell Counting Kit-8; ELISA, enzyme-linked immunosorbent assay.
