# Supplementary material for: Evaluation of the Antibacterial and Anti-Inflammatory Effects of a Natural Products-Containing Toothpaste
Source: Front Cell Infect Microbiol. 2022 Feb 10;12:827643. doi: 10.3389/fcimb.2022.827643 (PMC8867695; doi:10.3389/fcimb.2022.827643)
Supplement: Supplementary file 1 [file DataSheet_1.docx]

**Supplemental materials**

The compositions of the test toothpastes used in this study is showed in Table 1. The plant extracts were obtained from Jincun Herb, Co., Ltd. (Chengdu, China). The concentrations of the extracts were determined based on the effective concentrations of the 8 plant extracts in previous literature and their solubilities in toothpaste. The detailed information is showed in Table 2.

Table 1. The compositions of the test toothpastes.

| **Toothpaste** | **Active ingredients** | **Inactive ingredients** |
| --- | --- | --- |
| NPM-8-free toothpaste | 0.22% NaF | Sorbitol, glycerin, deionized water, hydrated silica, polyethylene glycol, sodium carboxymethyl cellulose, xanthan gum, sodium pyrophosphate anhydrous, disodium dihydrogen pyrophosphate, saccharin sodium, flavor, sodium cocoyl methyl taurate. |
| NPM-8-containing toothpaste | 0.22% NaF, NPM-8 (0.04% *Centella asiatica* extract, 0.016% *Polygonum cuspidatum* root extract, 0.016% *Scutellaria baicalensis* root extract, 0.008% *Camellia sinensis* leaf extract, 0.008% *Glycyrrhiza glabra* (*licorice*) root extract, 0.008% *Chamomilla recutita* (*Matricaria*) flower extract, 0.004% *Rosemary rosmarinus officinalis* leaf extract, and 0.004% *Salvia officinalis* (*Sage*) extract) | Sorbitol, glycerin, deionized water, polyethylene glycol, sodium carboxymethyl cellulose, xanthan gum, sodium pyrophosphate anhydrous, saccharin sodium, sucralose, flavor, mica, sodium cocoyl methyl taurate, perlite, hydroxyapatite, silica. |

Table 2. The effects and effective concentrations of the 8 plant extracts in previous studies, and concentrations used in NPM-8-containing toothpaste.

| **Plant extracts** | **Concentrations in NPM-8-containing toothpaste** | **Effects** | **Effective concentrations** | **References** |
| --- | --- | --- | --- | --- |
| *Centella asiatica* | 0.04%  (0.64 mg/ml) | Asiaticoside promotes type I collagen synthesis and osteogenic differentiation in human periodontal ligament cells | Asiaticoside: 10, 100 μM | (Bhaumik et al., 2012; Fitri et al., 2018; Soe et al., 2020). |
|  |  | Asiatic acid inhibits LPS-induced inflammatory response in human gingival fibroblasts | Asiatic acid : 25, 50,100 μM | - (Hao et al., 2017) |
| *Polygonum cuspidatum* root | 0.016%  (0.256 mg/mL) | Inhibit the viability, acid production and glucosyltranferase of *Streptococcus mutans* | MICs:  0.125–1 mg/ml | (Ban et al., 2010) |
| *Scutellaria baicalensis* root | 0.016%  (0.256 mg/mL) | Ameliorates the destruction of periodontal ligament via inhibition of inflammatory cytokine expression in animal study | 100 mg per kg animal | (Kim et al., 2018) |
| *Camellia sinensis* leaf | 0.008%  (0.128 mg/mL) | inhibits the growth of *S. mutans* and *P. gingivalis* | MIC  0.312 mg/mL | (Smullen et al., 2012)  (Araghizadeh et al., 2013) |
| *Glycyrrhiza glabra* (*licorice*) root | 0.008%  (0.128 mg/mL) | The antibacterial effect produced by *Glycyrrhiza glabra* (licorice) root extract on *S. mutans* was comparable to CHX while significantly higher in comparison with aqueous form and fluoride mouthwash. | MIC:  0.125 mg/mL | (Kamal et al., 2020) |
| *Chamomilla recutita* (*Matricaria*) flower | 0.008%  (0.128 mg/ml) | *Chamomile* extracts mouthwash has anti-inflammatory and antimicrobial actions similar to those of the chlorhexidine 0.12%, and was effective in reducing gingival bleeding in periodontal disease | MIC: 0.5% | (Batista et al., 2014) |
| *Rosemary rosmarinus* officinalis leaf | 0.004%  (0.064 mg/ml) | *Rosemary rosmarinus officinalis* leaf extracts inhibited glucosyltranferase activity, glucan production and plaque formation in vitro. | 0.025 mg/ml | (Smullen et al., 2012) |
| *Salvia officinalis* (*Sage*) | 0.004%  (0.064 mg/ml) | *Salvia officinalis* extracts inhibited glucosyltranferase activity, glucan production and plaque formation in vitro. | 0.025 mg/ml | (Smullen et al., 2012) |

**References:**

Al-Ani B.M., Owaid M.N., Al-Saeedi S.S.S. (2018) Fungal interaction between Trichoderma spp. and Pleurotus ostreatus on the enriched solid media with licorice Glycyrrhiza glabra root extract. Acta Ecologica Sinica 38:268-273. DOI: <https://doi.org/10.1016/j.chnaes.2017.08.001>.

Araghizadeh A., Kohanteb J., Fani M.M. (2013) Inhibitory activity of green tea (Camellia sinensis) extract on some clinically isolated cariogenic and periodontopathic bacteria. Med Princ Pract 22:368-72. DOI: 10.1159/000348299.

Ban S.H., Kwon Y.R., Pandit S., Lee Y.S., Yi H.K., Jeon J.G. (2010) Effects of a bio-assay guided fraction from Polygonum cuspidatum root on the viability, acid production and glucosyltranferase of mutans streptococci. Fitoterapia 81:30-4. DOI: 10.1016/j.fitote.2009.06.019.

Batista A.L., Lins R.D., de Souza Coelho R., do Nascimento Barbosa D., Moura Belem N., Alves Celestino F.J. (2014) Clinical efficacy analysis of the mouth rinsing with pomegranate and chamomile plant extracts in the gingival bleeding reduction. Complement Ther Clin Pract 20:93-8. DOI: 10.1016/j.ctcp.2013.08.002.

Bhaumik S.K., Paul J., Naskar K., Karmakar S., De T. (2012) Asiaticoside induces tumour-necrosis-factor-alpha-mediated nitric oxide production to cure experimental visceral leishmaniasis caused by antimony-susceptible and -resistant Leishmania donovani strains. J Antimicrob Chemother 67:910-20. DOI: 10.1093/jac/dkr575.

Cvetanović A., Švarc-Gajić J., Mašković P., Savić S., Nikolić L. (2015) Antioxidant and biological activity of chamomile extracts obtained by different techniques: perspective of using superheated water for isolation of biologically active compounds. Industrial Crops and Products 65:582-591. DOI: <https://doi.org/10.1016/j.indcrop.2014.09.044>.

Dziadek M., Dziadek K., Zagrajczuk B., Menaszek E., Cholewa-Kowalska K. (2016) Poly(ε-caprolactone)/bioactive glass composites enriched with polyphenols extracted from sage (Salvia officinalis L.). Materials Letters 183:386-390. DOI: <https://doi.org/10.1016/j.matlet.2016.07.077>.

Emima Jeronsia J., Ragu R., Sowmya R., Mary A.J., Jerome Das S. (2020) Comparative investigation on Camellia Sinensis mediated green synthesis of Ag and Ag/GO nanocomposites for its anticancer and antibacterial efficacy. Surfaces and Interfaces 21:100787. DOI: <https://doi.org/10.1016/j.surfin.2020.100787>.

Fitri A.R., Pavasant P., Chamni S., Sumrejkanchanakij P. (2018) Asiaticoside induces osteogenic differentiation of human periodontal ligament cells through the Wnt pathway. J Periodontol 89:596-605. DOI: 10.1002/JPER.17-0471.

Hao C., Wu B., Hou Z., Xie Q., Liao T., Wang T., Ma D. (2017) Asiatic acid inhibits LPS-induced inflammatory response in human gingival fibroblasts. Int Immunopharmacol 50:313-318. DOI: 10.1016/j.intimp.2017.07.005.

Hassan B., Tariq I.A. (2020) Phenolic compounds and hepatoprotective potential of Anastatica hierochuntica ethanolic and aqueous extracts against CCl4-induced hepatotoxicity in rats. J Tradit Chin Med 40:947-955. DOI: 10.19852/j.cnki.jtcm.2020.06.006.

Kamal D., Hassanein H., Akah M., Abdelkawy M.A., Hamza H. (2020) Caries Preventive and Antibacterial Effects of Two Natural Mouthwashes vs Chlorhexidine in High Caries-risk Patients: A Randomized Clinical Trial. J Contemp Dent Pract 21:1316-1324.

Kim M.H., Lee H., Choi Y.Y., Lee D.H., Yang W.M. (2018) Scutellaria baicalensis ameliorates the destruction of periodontal ligament via inhibition of inflammatory cytokine expression. J Chin Med Assoc 81:141-146. DOI: 10.1016/j.jcma.2017.04.013.

Quintana S.E., Villanueva-Bermejo D., Reglero G., García-Risco M.R., Fornari T. (2019) Supercritical antisolvent particle precipitation and fractionation of rosemary (Rosmarinus officinalis L.) extracts. Journal of CO2 Utilization 34:479-489. DOI: <https://doi.org/10.1016/j.jcou.2019.07.032>.

Smullen J., Finney M., Storey D.M., Foster H.A. (2012) Prevention of artificial dental plaque formation in vitro by plant extracts. J Appl Microbiol 113:964-73. DOI: 10.1111/j.1365-2672.2012.05380.x.

Soe H., Luckanagul J.A., Pavasant P., Jansook P. (2020) Development of in situ gel containing asiaticoside/cyclodextrin complexes. Evaluation in culture human periodontal ligament cells (HPLDCs). Int J Pharm 586:119589. DOI: 10.1016/j.ijpharm.2020.119589.
